# Analyzing the Photoprotection Efficiency of Sunscreens Containing Antioxidants under Disinfection Conditions

**DOI:** 10.3390/antiox10111720

**Published:** 2021-10-28

**Authors:** Robert Sotler, Metka Adamič, Kristjan Jarni, Raja Dahmane, Polonca Trebše, Mojca Bavcon Kralj

**Affiliations:** 1 Faculty of Health Sciences, University of Ljubljana, Zdravstvena pot 5, 1000 Ljubljana, Slovenia; Robert.sotler@zf.uni-lj.si (R.S.); raja.gosnak@zf.uni-lj.si (R.D.); 2 Dermatology Metka Adamič, Vilharjeva 25, 1000 Ljubljana, Slovenia; info@metkaadamic.com; 3 Department of Forestry and Renewable Forest Resources, Biotechnical Faculty, University of Ljubljana, Jamnikarjeva ul. 101, 1000 Ljubljana, Slovenia; kristjan.jarni@bf.uni-lj.si

**Keywords:** ultraviolet radiation, UV filter, benzophenone-3, trans-resveratrol, β-carotene, chlorinated products

## Abstract

Sunscreens ensure thorough protection against sunburn. The delivery of UV filters into the stratum corneum and viable epidermis could be reduced by the use of antioxidants (such as β-carotene and trans-resveratrol, alone or combined). The presence/absence of antioxidants (trans-resveratrol and β-carotene) in formulations containing benzophenone-3 (UV-filter) and their efficiency under disinfection and neutral conditions are studied and compared. The trial was conducted on 38 people. The prepared ointments were applied to the participants’ forearms, irradiated and monitored by reflectance colorimetry after 0, 4, 6, and 8 min. Descriptive statistics were used to describe the skin color’s main characteristics and the F-ratio was used to test overall differences. The ointments containing antioxidants and benzophenone-3 were the most efficient, followed by those with benzophenone-3 alone. It was proven that photoprotection with benzophenone-3 is still effective, despite the formation of its chlorinated products. Due to the short time of exposure to disinfecting conditions, it could be assumed that benzophenone-3 was only partially chlorinated. This clinical study demonstrated that formulations containing antioxidants are likely to be more suitable for protecting skin against UVB irradiation than a UV filter alone.

## 1. Introduction

Sunscreens are primarily designed to protect the skin from the harmful effects of solar ultraviolet radiation (UV irradiation). Chronic excessive and frequent exposure to UV irradiation can cause the occurrence of malignant skin changes [1,2,3]. Following the increasing awareness of skin cancer, photoprotection with sunscreens has been widely advocated. The molecules or molecular complexes added to sunscreen as basic constituents can absorb, reflect, or scatter UV photons. Compounds that absorb UV light are usually called UV filters, in addition to inorganic pigments, which reflect UV light in particular [4]. Because UV light features a broad spectral range, 400–290 nm (UVA and UVB), no single compound is capable of protecting against exposure to the whole spectrum, since their absorption peaks are much narrower. Therefore, a combination of several compounds that covers the whole area is usually applied. The European Union (EU) currently allows 26 organic substances, while others, which are treated as biological agents, are allowed without prescription and regulation [5] in countries around the world, such as Japan and the U.S.

Testing the sun protection ability of sunscreens represents an important part of the optimization process to ensure thorough protection of the skin against sunburn. The level of sun protection has traditionally been estimated using the sun protection factor (SPF) test, which utilizes the erythemal response of the skin to UVB radiation. The method is based on determining the minimum erythematous dose (MED), defined as the smallest amount of energy required for triggering erythema, in areas of protected and unprotected skin [6]. Color assessment of skin by visual inspection alone may be precise for a given individual, although the comparison between colors is only possible when they are viewed simultaneously. The limitations of visual observations may be overcome by color-order systems and by instrumental measurements using either reflectance spectrophotometry or reflectance colorimetry following the Commission Internationale de l’Eclariage (CIE) recommendations [7].

To prolong the duration of SPF, compounds with antioxidant properties are added to sunscreens. Since antioxidants include a range of low-molecular weight compounds, they provide a well-functioning network system responsible for cell protection. The balance between the formation of radicals and their removal is extremely important for the overall optimal functioning of the cell. If the equilibrium breaks down, excessive ROS are formed, which may cause damage to the cell’s components. The cell’s protection capacity is consequently defined as the cell’s ability to neutralize ROS through the action of the antioxidant defense system. UV filters, in general, should not permeate skin cells. They should not interfere with systemic circulation. However, the UV filter, benzophenone-3 (BP-3), was detected in the plasma and urine of humans (women more than men; [8]) and tested animals (rats) [9,10] after the topical application of sunscreens. This phenomenon leads to poor skin photoprotection and induces photosensitivity due to the cutaneous penetration of UV-filters in viable skin layers causing toxic or allergic reactions [11]. The application of antioxidants, such as β-carotene and trans-resveratrol, alone or combined, reduced the delivery of UV filters into the stratum corneum and viable epidermis [11,12].

In fact, in addition to questionable health perspectives, several recent studies showed that the use of UV filters has caused environmental issues, as persistent transformation products are formed in the aquatic biosphere and, in this way, they affect public health in the long term (three major metabolites were found in organisms in vivo studies, i.e., benzophenone-1 (BP-1), benzophenone-8 (BP-8), and 2,3,4-trihydroxybenzophenone (THB)) [13]. The transformation product BP-1, unfortunately, shows greater estrogenic potency in vitro, with a longer biological half-life than the parent compound BP-3. The acute toxicity of BP-3 and other UV-filters on Daphnia magna increases with the increasing log Pow of the compound [14], causing a variety of toxic reactions in corals and fish, ranging from reef bleaching to mortality [15]. It has now been detected across all the compartments of the aquatic ecosystem (ambient freshwater–125 ng L^−1^; seawater–577.5 ng L^−1^, wastewater influent–10,400 ng L^−1^; [13]), with human recreational activities (swimming in waters) and wastewater treatment plants being its major sources.

The chemical and microbiological control of swimming waters affected by undesirable biological matter (e.g., skin, sweat, urine or fecal matter) is maintained through disinfection, usually chlorine (as gas or bleach). The results are numerous disinfection by-products (DBPs), including chloramines (referred to as combined chlorine), trihalomethanes (THMs) and other chlorinated aromatic compounds, all associated with health effects [16]. Since sunscreens are used mainly in leisure or training activities, such as swimming in the sea or open swimming pools, where comprehensive protection is needed, the transformation of original sunscreen and antioxidants in these locations is expected. Due to the complexity of swimming pool water chemistry, human exposure and the potential toxicology risks arising from these activities, health and environmental perspectives are still poorly addressed.

It is currently understood that two types of reactions occur in swimming waters: (a) direct photolytic reactions [17], and (b) the chlorination of aromatic rings or side chains due to the presence of active chlorine medium (swimming pool water, seawater) [18,19]. The formation of halogenated DBPs in chlorinated waters is inevitable, especially when UV filters possess double bonds, phenolic, keto, or amino moieties [20]. In both cases, parent UV filters, providing UV protection, degrade and may lose their photoprotective role. Several studies reported various reactions of UV filters in swimming waters under chlorinated conditions, leading mainly to the formation of chlorinated DBPs, with an emphasis on the determination of primary chlorinated derivatives, or their further degradation [21,22,23,24,25]. Photostability studies showed that dichloro-derivatives in chlorinated waters are less stable than the parent compound, which is not the case for monochloro-derivatives (formed initially) [22] in the case of BP-3. Antioxidants, added to sunscreens, may react under disinfection conditions as well. Over 80 transformation products of resveratrol were identified using GC-HRMS and UPLC-HRMS, with chlorinated phenols and biphenyls found to be the most relevant among them [26]. Furthermore, β-carotene-type terpenoids mostly form trihalomethanes (CHBrCl_2_, CHBr_2_Cl, CHBr_3_) as final disinfection products, most likely through haloform reactions on methyl ketone groups in the complex suites of reaction intermediates. Several other products, such as β-cyclocitral, trans-β-ionone-5,6-epoxide, and β-ionone have also been identified [27]. In general, the benefits of using a combination of antioxidants in sunscreens were demonstrated by showing that using trans-resveratrol, β-carotene and the studied UV-filters (octyl methoxycinnamate, avobenzone, octocrylene, bemotrizinole, octyltriazone) led to more photostable formulations, which in turn implies better safety and efficacy [28].

The controversies over sunscreen safety and toxicity in the case of BP-3 were discussed in a previous study [29]. The benefits of UV protection are undoubted; however, with the increased usage of sunscreens by the public, continuous monitoring of the overall safety of future products is clearly needed [30]. The chlorination or degradation of photoprotective compounds and antioxidants may lead to the loss of their photoprotective role.

Our clinical study aimed to investigate skin protection efficiency using different formulations containing one UV-filter (BP-3) and two antioxidants (trans-resveratrol and β-carotene) under various conditions, including disinfection conditions. It was conducted to answer two questions: (1) the difference between the effects of UV irradiation on skin protected with clothes and exposed skin (not protected with clothes); and (2) the difference between the effects of UV irradiation on exposed skin (positive control) and ski, protected with ointments in chlorinated and non-chlorinated waters, measured with reflectance colorimetry.

## 2. Materials and Methods

### 2.1. Materials

The chemical components and the preparation of the tested ointments were as follows: almond oil (All Organic Treasures, Wiggensbach, Germany) (30 wt.%) was melted, and glyceryl stearate (SE, Caesar & Loretz, Hilden, Germany) (5 wt.%), cetearyl alcohol (Aliacura, Scheinfeld, Germany) (1 wt.%), glyceryl caprylate (Herbana, Ljubljana, Slovenia) (0.5 wt.%), and benzophenone-3 (Sigma-Aldrich, Darmstadt, Germany) (5 wt.%) were added to the oil in a water bath. The antioxidants β-carotene (Sigma-Aldrich, Darmstadt, Germany) (1 wt.%), or trans-resveratrol (Sigma-Aldrich, Acros Organics) (1 wt.%) were then added slowly to ensure that the lipid phase was completely molten and homogeneous in appearance. Demineralized water (58 wt.%) was heated to the same temperature (60–65 °C) and added to the lipid phase during homogenization at the fastest speed. Homogenization took place for another 3 to 5 min in a cold bath until the ointment was cooled to about body temperature. Xanthan (Organic Factory, Vailate, Italy) (0.3 wt.%) was suspended in glycerol (All Organic Treasures, Wiggensbach, Germany)) (5 wt.%) and mixed thoroughly. Next, a preservative, dehydroacetic acid (Sigma-Aldrich, Darmstadt, Germany) (8 wt.%) in benzyl alcohol (Sigma-Aldrich) was added, mixed thoroughly, and acidified to pH 5 to 5.5 with lactic acid (90%, Caesar & Loretz Hilden, Germany).

### 2.2. Methods

#### 2.2.1. Study Design

For this study, a controlled clinical trial was conducted in which an area of the forearm was irradiated with a UV lamp on 38 volunteer subjects (aged between 20 and 60; male: 10; female: 28). Before the beginning of the study, an agreement from the Slovenian National Medical Ethics Committee was issued (16 May 2018, No. 0120-368/2017/5). The clinical study was conducted between June and August 2019, in the laboratory of the Faculty of Health Sciences, at the University of Ljubljana, under controlled conditions.

The volunteers provided informed consent and completed a questionnaire for the self-assessment of skin type and sensitivity, and the self-determination of the suitability criteria for their participation in the clinical study. According to the Fitzpatrick scale (1988), we decided to study average-sensitive white skin, the second and third skin phototypes. Phototype II usually burns, tans minimally (light-colored but darker than fair); phototype III sometimes experiences mild burns and tans to a golden honey color [31]. There were no differences observed between phototypes II and III, since they were both average-sensitive white skin. The analyzed group was thus homogenous. Allergic reactions were one of the exclusionary criteria. Photoallergy resembles contact hypersensitivity. The only difference is that chemicals producing photoallergic reactions require activation by light to provoke the immune photoallergic response [32]. For this reason, those allergic to benzophenone was excluded from patch testing before the clinical trial. 

#### 2.2.2. Assay Methods and Conditions

The clinical trial was conducted in four stages. In the first stage, before the experiment, the irradiation area of the skin’s surface was carefully examined. The questions in the questionnaire for self-assessment, including information regarding volunteers’ potential pregnancy, skin diseases, skin changes, allergic reactions, and age, were exclusionary. The questions about skin type, sensitivity, and self-determination of the suitability criteria for participation were not exclusionary. Following the established scale [31], the participants with skin phenotype II and III (both phenotypes are defined for persons with average-sensitive skin color) were chosen.

In the second stage, the left forearm was chosen as the irradiation field for determining the minimal erythema dose – MED. For this purpose, the forearm was shielded from irradiation with a custom-made glove, apart from the irradiation area, which comprised three squares cut into the glove. For anatomical reasons, the surface of the squares was 3 × 3 cm (9 cm^2^), as the length of the adult forearm is approximately 20 cm, to ensure that all the test participants’ skin surfaces were exposed to a similar area (9 cm^2^) during the experiment. The squares were irradiated over different periods (the first square was uncovered for the entire 8 min, the second for 6 min, and the third for 4 min).

The third stage took place 20 to 28 h after the first irradiation. For the MED evaluation, all the irradiation fields were thoroughly examined, measured, and photographed. The results were entered on a separate form and submitted with photo documentation for evaluation by two dermatologists.

**Figure 1:**
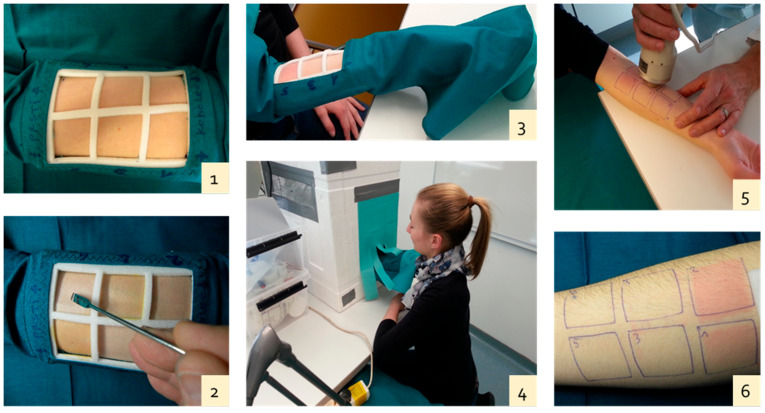
The irradiation field with a custom-made glove with six cut squares (**1**), the application of individual ointment (**2**), the position of six squares 3 × 3 cm (9 cm^2^) on the adult forearm, (**3**) the glove use, (**4**) the tailor made irradiation chamber, (**5**) the skin color measurement, (**6**) the differences in skin coloration.

In the fourth stage, after receiving a medical evaluation from the two dermatologists, who had prior knowledge of the irradiation time, the tested volunteers were again invited and subjected to a controlled clinical study. The irradiation field was again protected using a custom-made glove, this time with six cut squares (Figure 1). A constant irradiation time was applied for all the squares determined by the dermatologists, under the following conditions:

- the first square (positive control): the irradiated area remained free of ointment;
- the second square: a neutral ointment, without a UV filter or antioxidants, was applied;
- the third square: an ointment with a UV filter BP-3 was applied;
- the fourth square: an ointment with a UV filter BP-3 was applied. Before applying the ointment, the skin zone was exposed to disinfection conditions similar to those in swimming pool water for 1 min;
- the fifth square: an ointment with both a UV filter BP-3 and an antioxidant, β-carotene, was applied;
- the sixth square: an ointment with both a UV filter BP-3 and an antioxidant, trans-resveratrol, was applied.

#### 2.2.3. UVB Irradiation and Measurement Techniques

A targeted scientific experiment on the target population of subjects was conducted to find suitable and effective means of exposing human skin to UVB radiation conservatively and protectively. The irradiation was carried out with a portable UV lamp with the following technical characteristics: dual 302 nm wavelength light tube lamp, 8 W, 2000 μW cm^−2^ (Cole-Parmer, Chicago, IL, USA), with an energy density of 1 J cm^−2^ at an irradiation wavelength of UV-B radiation between 280 and 320 nm. 

#### 2.2.4. Skin Color Measurements

A portable colorimeter (Konica Minolta Chroma Meters CR-410 [Tokyo, Japan]) was used to measure skin color using the guidelines for skin color measurement and erythema [32]. This colorimeter records the change in developing erythema and skin tone in numbers. According to the CIE (Commission Internationale de l’Eclairage; English: International Commission on Illumination) for measuring the color of surfaces, with the results given in L* a* b*, parameters (color spaces for measuring object colors). They can also be applied to natural UV tanning and the artificial chemical tanning of human skin [33,34]. The skin redness is observed as the numerical decrease in the L* parameter and the increase of the b* parameter due to higher levels of absorbed and lower levels of reflected green light, according to Clarys et al. [35]. The a* and b* coordinates may be converted into more useful polar coordinates, defining the hue angle [34], which is more appropriate for measuring skin tone. The CIE’s recommended hue angle is the psychometric correlation of the visually perceived attribute expressed in degrees, where hue = 0° means red color and hue = 90° means yellow color. It is calculated as “Arcus tangents” of the quotient between a* and b* coordinates: h° = arctan (b*/a*).

#### 2.2.5. Statistical Analysis

The analysis included 38 people who were exposed to UV irradiation of the skin. The skin color was checked after 0, 4, 6, and 8 min. Descriptive statistics, such as arithmetic means and standard deviation, were used to describe the skin colors’ statistical characteristics after UV irradiation. As all the participants were included in all the experimental conditions, one-way repeated-measures ANOVA was used to determine how the time of UV exposure affected skin color. The F-ratio used in ANOVA is a ratio between the average variability in the data that a given model can explain and the average variability that is not explained by the same model. The F-ratio is used to test overall differences between group means in the experiment. The assumption of sphericity was checked through Mauchly’s test. 

To compare the effects between different exposure times, the “repeated contrast” test was used, where the results of exposure (e.g., after 6 min) were compared with the results of the previous measurement (e.g., after 4 min). All the calculations were performed using IBM SPSS Statistics software.

## 3. Results

### 3.1. Determination of the Minimal Erythema Dose (MED)

The calculated numerical values expressed in degrees (the hue angle) of the 38 volunteers are collected in Table 1.

Before comparing the skin color with the duration of UV exposure (Table 2), Mauchly’s test of sphericity was calculated. It was performed in order to determine whether the differences were statistically significant (violation of sphericity). The Mauchly’s test showed that the assumption of sphericity was not violated χ2(5) = 11.41, *p* > 0.05, and it proved that the variances of the differences between all the possible pairs of within-subject conditions were equal.

The results (summarized in Table 2) showed that skin color was significantly affected by the duration of UV exposure (Pillai’s Trace (V) = 0.59, F (3, 35) = 16.92, *p* < 0.001). The first four minutes (difference from 0 min to 4 min) of UV irradiation revealed no significant change in skin color (F = 0.14, *p* > 0.05). However, the color of the skin after 6 min of UV irradiation was significantly redder (hue angle decreased, F = 4.50, *p* < 0.05) as compared to 4 min of irradiation. When the irradiation time was extended from 6 min to 8 min, the change in skin color was even more pronounced, and there was a highly statistically significant difference (a significant decrease in hue angle; the highest values in our experiment F = 27.91, *p* < 0.001; Table 2) compared to the previous period. According to the collected results, the MED dose was set to 8 min. 

### 3.2. Comparison of the Effect of the Applied UV Filter and Antioxidants

In the first phase of the clinical study, a comparison between covered and uncovered skin exposed to UV irradiation was tested. It was conducted on 26 patients whose skin was exposed to a MED time of 8 min. The six squares were irradiated with the same exposure time determined for each individual in the MED pre-phase. Simple descriptive statistics, arithmetic means, and standard deviations were used to describe the main features of the skin colors and a paired sample test was used to compare the effects between covered skin (protected with the custom-made glove) and UV -rradiated skin.

The hue angle decreased in the case of covered skin (56.1°) (Table 3 and Figure 1 skin around the six squares) compared to a positive control (51.9°) (Table 3 and Figure 1–square 1: the irradiated area remained free of ointment). The hue angle decreased even more in skin covered with a neutral ointment (49.6°) (Table 3 and Figure 1–square 2: neutral ointment applied on the skin). This indicated that significant measurable redness developed after UV exposure. There was even more pronounced erythema in the second square (placebo neutral ointment). Interestingly, square one was generally less red than the second, indicating that one of the placebo components may have accelerated erythema.

Surprisingly, a statistical difference with covered skin was noted in the case of an ointment containing BP-3, even when in contact with chlorinated water (Table 3: 57.6°; 57.4°, respectively). This result confirms numerically that ointments containing UV-protective filters, such as BP-3, are effective blockers of sunburn. Another observation was that the ointments containing UV filters (BP-3) and antioxidants (β-carotene and trans-resveratrol) were even more successful at skin protection as there were significant shifts towards paler skin coloration (Table 3: 58.7; 58.0, respectively).

The paired sample test enabled us to compare the differences between the hue angles of the skin tone. The first pair test showed a significant difference between the redness of uncovered and unshielded skin (marked as the positive control) and the covered skin. Similarly, the neutral ointment applied on the skin could not prevent erythema, since significant redness (hue angle = −6.5°) was observed (Table 4). The skin shielded by the UV filter BP-3 was wholly protected, and its tone was identical to the covered skin, even if the ointment was later in contact with chlorinated water (the 4th pair). It can be assumed that due to the short time of exposure to disinfectant, applied only to the skin’s surface, not all UV filters in sunscreen were chlorinated.

The addition of antioxidants (β-carotene – the 5th pair, and trans-resveratrol – the 6th pair) caused significant shifts toward yellow skin tone (hue angle = +2.6° and hue angle = +2.0°; respectively). The ointment with a UV filter and antioxidants protected the skin more efficiently than the ointments with UV filter only. The skin color was even paler than the covered skin.

The differences between the hue angles of the uncovered skin (the positive control) and the skin tones in the case of skin treated with ointments showed that the UV filter BP-3 was efficient at protecting the skin against sunburn. Positive shifts in skin tones indicated that slight redness was found in all four cases when the ointment contained the UV filter. The ointments containing the two antioxidants (hue angle = +6.7° − for β-carotene; and hue angle = +6.1 − for trans-resveratrol) were the most efficient, followed by the ointments that contained UV filters only (hue angle = +5.6° − for UV filter; and hue angle = +5.4 − for UV filter on skin exposed to chlorinated water) (Table 4).

## 4. Discussion

The role of antioxidants in sunscreens has previously been reported in a study by Gaspar and Campos [36], where the photostability of various formulations containing UV-filters in combinations and vitamins A, C, and E were investigated. It was demonstrated that the formulation containing BP-3 and vitamins A, C, and E reduced the UVA/UVB-absorption ratio after 30 min of irradiation. The study also demonstrated that the presence of vitamins reduces skin irritation. This is in accordance with the results obtained in our study.

Several studies deal with the identification of chlorinated products of UV filters in swimming pool waters; however, there is no information about degradation (chlorination) products in sunscreens and the subsequent potential loss of photoprotection and/or skin allergies. Our previous studies on the chlorination of BP-3 in water have demonstrated the formation of several chlorinated products (3-chlorobenzophenone, 5-chlorobenzophenone, and 3,5-dichlorobenzophenone); however, their effect on skin was not tested at that point [22]. When a substance comes into contact with a chlorine-based disinfectant, the chlorination reaction takes place immediately [22]. We assume that a similar reaction occurs in the thin film of sunscreen on the skin. The chemical characterization of products has shown that they absorb in the same range of light as the parent UV filter [37], and for this reason, we assumed the loss of the photoprotective role would not take place. In fact, the formed benzophenone-3 chlorination products were photostable (more than 95% of the initial concentration) during the irradiation periods (8 min, 6 min, and 4 min). Possible allergic reactions that might occur due to the formation of chlorinated products (due to their high stability, especially 3- and 5-chlorobenzophenone, and low solubility in water) [22] cannot be excluded. The protective role of antioxidants in disinfection conditions has been supported by another study, dealing with the behavior of resveratrol in chlorinated waters. It reported the formation of more than 80 products, expressing resveratrol’s protective role and its high potential for acting as a scavenger of reactive oxygen species (ROS) in sunscreens [26], and confirms the comparison of pairs 5 and 6 in Table 4. 

Since BP-3 features some drawbacks, several derivatives (BP-3 carbonate, BP-3 carbazole, BP-3 phenylamine, and BP-3 methoxy-phenylamine [38]) and formulations with the addition of cyclodextrin complexes [39] and chitosan-coated nanocapsules [40] were developed as a promising strategy for improving the photoprotective effect of benzophenones. The addition of antioxidant molecules is likely to benefit UV filters by protecting against UV degradation and other in vivo skin effects [41].

Among the challenges and concerns associated with topical sunscreen formulations, in this study, we explored the concern related to the formation of chlorinated products, formed through their reaction with disinfection agents. Namely, bathing in swimming pools or thalassotherapy pools, (where brominated and iodinated products are also formed) demands strict disinfection conditions to secure the microbiological safety of pool waters. There are numerous research data on disinfection products formed under such conditions; however, public health and safety guidelines on this matter are lacking. For now, several parameters, such as the photostability of organic filters, broadening the effectiveness, the addition of active ingredients, and improving cosmetic and sensory aspects, are involved [5,21], but there is no regulation regarding substances occurring under disinfection conditions and their effects on human health and the environment. Recently, promising results were obtained from a combined UV degradation and chlorination study of BP-3, where chlorinated transformation products (TPs) exposed to UV light were less toxic to Vibrio fischeri than chlorinated TPs that were not exposed to UV light. The findings imply that the additional oxidation of TPs occurs through the reactive radicals produced during the UV/chlorination reaction [42]. The experiments reported herein demonstrate similar effects of the UV irradiation of chlorinated BP-3 on human skin. For this reason, additional studies on the effects of chlorinated products of UV filters (as well as other components of sunscreens) on the skin should be performed and included in the risk assessment documents for each formulation used in the production of sunscreens.

## 5. Conclusions

In summary, this clinical study showed that formulations containing antioxidants are likely to be more suitable for protecting skin against UVB irradiation than UV filters alone. Formulations with UV filters and antioxidants (trans−resveratrol and β-carotene) reduced skin irritation. In the case of BP-3 in the presence of chlorinated water, photoprotection was not lost, despite the formation of chlorinated products.

## Figures and Tables

**Table 1:** Descriptive statistics of skin color in regards to the duration of UV exposure (N = 38).

Time of Exposure [min]	Hue Angle–Mean Value [°]	Std. Deviation [°]
0	56.0	6.3
4	56.5	7.6
6	54.5	7.7
8	48.5	8.0

**Table 2:** Comparison of skin color (F-ratio) across four periods of irradiation.

Comparisons	F-Ratio	Significance
Observation at 0 min vs. 4 min	0.145	0.706
Observation at 4 min vs. 6 min	4.503	0.041
Observation at 6 min vs. 8 min	27.912	0.000

**Table 3:** The descriptive statistics of the main features of skin color after 8 min UV irradiation (N = 26 volunteers).

Descriptve Analyses	Mean (Hue Angle)	Std. Deviation
Covered skin	56.1	6.0
Positive control	51.9	8.2
Neutral ointment without additives	49.6	7.2
UV filter–benzophenone 3	57.6	5.9
UV filter–benzophenone 3 + chlorinated water	57.4	7.1
UV filter–benzophenone 3 + β-carotene	58.7	6.4
UV filter–benzophenone 3 + trans-resveratrol	58.0	5.9

**Table 4:** The paired sample test – the comparison between skin squares and the covered/uncovered skin in the clinical study.

Comparisons	Mean ± Std. Dev.	95% Confidence		Sig. (2-Tailed)
		Lower	Upper	
1st Pair: Controls comparison				
Uncovered–Covered skin	−4.1 ± 7.0	−7.0	−1.3	0.006
2nd Pair: Neutral ointment without additives				
Covered skin	−6.5 ± 7.6	−9.5	−3.4	0.000
Uncovered skin	−2.3 ± 3.9	−3.9	−0.7	0.005
3rd Pair: BP-3				
Covered skin	1.5 ± 4.1	−0.2	3.1	0.075
Uncovered skin	5.6 ± 5.9	3.2	8.0	0.000
4th Pair BP-3 with the addition of chlorinated water				
Covered skin	1.3 ± 4.9	−0.7	3.3	0.188
Uncovered skin	5.4 ± 6.5	2.8	8.1	0.000
5th Pair BP-3 with the addition of β-carotene				
Covered skin	2.6 ± 3.8	1.1	4.1	0.002
Uncovered skin	6.7 ± 6.7	4.0	9.5	0.000
6th Pair BP-3 − trans-resveratrol				
Covered skin	2.0 ± 4.4	0.2	3.7	0.031
Uncovered skin	6.1 ± 6.3	3.6	8.6	0.000

## Data Availability

The data presented in this study are available upon request at robert.sotler@zf.uni-lj.si. The data are not publicly available due to The General Data Protection Regulation (GDPR).
